# Laparoscopic sacrocolpopexy posthysterectomy: intraoperative feasibility and safety in obese women compared with women of normal weight

**DOI:** 10.1007/s00192-019-03888-y

**Published:** 2019-02-27

**Authors:** Charlotte Mahoney, Georgina Scott, Lucy Dwyer, Fiona Reid, Karen Ward, Anthony Smith, Rohna Kearney

**Affiliations:** 1 Institute of Human Development, Faculty of Medical & Human Sciences, University of Manchester, Manchester, England; 2 The Warrell Unit, St Mary’s Hospital, Manchester University Hospitals NHS Foundation Trust, Manchester Academic Health Science Centre, Manchester, M13 9WL England; 3 MRCOG, Department of Urogynaecology, The Warrell Unit, St Mary’s Hospital, Oxford Road, Manchester, M13 9WL UK

**Keywords:** Obesity, Vault prolapse, Laparoscopic sacrocolpopexy, Safety

## Abstract

**Introduction and hypothesis:**

Our aim was to determine the intraoperative feasibility and complication rate of laparoscopic sacrocolpopexy (LSC) in overweight and obese women compared with women of normal weight.

**Methods:**

This was a retrospective observational cohort study (Canadian Task Force classification II-2) conducted at a tertiary urogyaenocology unit evaluating 119 women who underwent LSC between March 2005 and January 2013.

**Results:**

Body mass index (BMI) was classified as normal (22.89 ± 1.55), overweight (27.12 ± 1.40) and obese (33.47 ± 3.26) according to the World Health Organisation (WHO) classification. There was no difference in intraoperative complication rates for bladder, bowel, ureteric or vascular injury; haemorrhage; conversion to laparotomy; or anaesthetic complications for normal weight, overweight or obese women. Similarly there was no difference in operating time, duration of anaesthetic or hospital stay between BMI class (*p* = 0.070, *p* = 0.464, *p* = 0.898, respectively) postoperative or mesh complication rates. At 6-months’ follow-up, there was no difference in Patient Global Impression of Improvement scale (PGI-I) (defined as very much better or much better) between normal weight, overweight and obese women (76.9, 72 and 65.4%, *p = .669*) or objective cure using the Pelvic Organ Prolapse Quantification (POP-Q) examination (*p = 0.402*).

**Conclusions:**

LSC is feasible, with equivalent intraoperative complication rates for normal weight, overweight and obese women when performed by experienced laparoscopic urogynaecologists. Given the benefits of a laparoscopic approach in obese women, the authors suggest they should be offered LSC as an option to treat vault prolapse when surgical management is being considered.

## Introduction

Posthysterectomy vaginal vault prolapse is a common presentation to the urogynaecology clinic, with an incidence of 11.6% following hysterectomy for pelvic organ prolapse (POP) and 1.8% for benign causes [1]. Obese women are more likely to develop vault POP than women of normal weight [2, 3]. The rate of obesity is increasing, with predictions suggesting two thirds of the world’s population will be overweight or obese by 2030 [4]. On this basis, it is reasonable to expect an increase in the number of obese women with vault prolapse seeking surgery.

Abdominal sacrocolpopexy (ASC) is the standard surgical treatment option for vault prolapse; however, laparoscopic sacrocolpopexy (LSC) has been shown to have similar outcomes, lower complication rates and shorter hospital stay than the open approach [5, 6]. Yet there remains a reluctance by some urogynaecologists to offer LSC to obese women with vault POP [7], which may be due to perceived difficulty in performing the procedure safely in obese women. Medium-term outcomes of purely LSC in obese women have been described in only one study to date: Thubert et al. evaluated outcomes and safety of LSC in obese and nonobese women, reporting clinical equivalency [8]. Limitations of their study were the combination of normal weight and overweight women into one nonobese group and a short follow-up period of 2 months.

Despite the obesity epidemic, there remains a paucity of published data on outcomes or complications rates for prolapse surgery in obese women. There is now a growing need for better safety and efficacy data on surgery in this group of women. Therefore, the primary aim of this study was to retrospectively evaluate the intraoperative feasibility and complication rates of LSC (excluding robotic sacrocolpopexy) in normal weight, overweight and obese women attending a tertiary urogynaecology service. The secondary aim was to evaluate functional and anatomical outcomes 6 months postoperatively.

## Methods

### Patients and procedures

A single-centre, retrospective cohort study was performed to evaluate the outcomes and adverse events following insertion of a lightweight mesh, Restorelle^®^, in women undergoing LSC (manuscript accepted for publication in European Journal of Obstetrics and Gynecology). The study excluded sacrohysteropexy, as it was designed to evaluate a mesh specifically designed for sacrocolpopexy. Secondary analysis was performed to evaluate the efficacy and safety of LSC in obese women compared with those with a normal body mass index (BMI). All women who underwent LSC between March 2005 and January 2013 were invited to participate and provided informed consent. During this time, operating surgeons routinely offered LSC to all women presenting with vault prolapse defined as point C at stage 2, or stage 1 with a large concomitant anterior- or posterior-compartment POP, irrespective of their BMI. Contraindications to a laparoscopic procedure included previous major intra-abdominal surgery with significant intra-abdominal scarring preventing access to the sacral promontory, and respiratory or cardiovascular disease leaving women unable to tolerate a general anaesthetic. Inclusion criteria for secondary analysis were surgery for LSC, BMI at the time of surgery recorded in the case records and patient willingness and ability to provide informed consent and attend follow-up examination. Women were excluded if inclusion criteria were not met. All operations were performed in a tertiary unit by one of three subspecialist surgeons trained in laparoscopic urogynaecology.

### Ethical approval

The study was approved by the North-West Greater Manchester Central Research Ethics Committee (reference 12/NW/0277) and registered with Current Controlled Trials (ISRCTN19907894). Informed consent was obtained from all individual participants in the study.

### Surgical technique

The same technique was used for all women, regardless of BMI. LSC was performed under general anaesthesia, and all women received antibiotic prophylaxis. Women were placed in the Lloyd-Davies position and a Foley catheter inserted. Three trocars were used: one primary umbilical 10-mm cannula and two lateral (one 5 mm and one 11 mm) trocars. The sacral promontory was identified, the prevertebral parietal peritoneum dissected to expose the retroperitoneal fat and the retroperitoneal fat dissected to expose the anterior vertebral ligament. The peritoneal incision was then extended towards the rectosigmoid. The bladder was dissected from the anterior vaginal wall and the peritoneum dissected from the posterior vaginal wall. The dissection was extended in cases of a large rectocele or cystocele to facilitate extension of mesh placement. The two arms of a Y-shaped piece of mesh were sutured to the anterior and posterior walls of the vagina using polydioxanone (PDS), with a minimum of four sutures on each. The vault was positioned tension free at the level of the ischial spines and the mesh fixed to the sacral promontory using Protack staples. The peritoneum was then closed using a continuous running suture.

### Study protocol

Demographic data on age, smoking status, parity and mode of any deliveries, preoperative comorbidities, prolapse symptoms, prior conservative management and previous prolapse or incontinence surgery was collected. Preoperative BMI was used to group women into BMI class according to the World Health Organisation (WHO) classification: normal weight = 18.5–24.9 kg/m^2^; overweight = 25.0–29.9 kg/m^2^; obese ≥30 kg/m^2^ [9]. Complications were recorded using the International Continence Society/International Urogynaecological Association (ICS/IUGA) classification of complications related directly to the insertion of prostheses (meshes, implants, tapes) or grafts in female pelvic floor surgery [10]. Paper case notes and electronic data systems were hand-searched for information regarding operating time, duration of anaesthetic, anaesthetic complications and duration of in-patient stay.

Preoperatively, all women underwent a Pelvic Organ Prolapse Quantification (POP-Q) system examination. Postoperatively, they underwent POP-Q grading and an assessment for mesh palpability. All scores were converted to their corresponding stages using the system described by IUGA/ICS [11]. Postoperative assessment was routinely conducted by an independent health-care practitioner, who was not the operating surgeon, to limit bias. Women completed a Patient Global Impression of Improvement (PGI-I) questionnaire for urogenital prolapse to assess subjective improvement after treatment [12]. All data were collected by one researcher and cross-checked by one of two other researchers for standardisation.

### Outcome measures

The primary outcome measure of safety was evaluated using perioperative complication rates, duration of operation and anaesthetic and duration of in-patient stay. Secondary outcomes were patient-reported success rate using PGI-I, defined as very much better, much better and objective cure (defined as prolapse stage 0 or 1 on POP-Q examination). Data were analysed using two definitions of recurrence: vault POP stage ≥2, and point C above the level of the midvagina (defined as half the total vaginal length).

### Statistical analysis

Outcomes were stratified according to BMI and statistical analysis performed using Stata version 15.0 (StataCorp, TX, USA). Analysis of variance (ANOVA) was used to test for age, operating time and duration of anaesthetic. Kruskal-Wallis equality of populations rank test was used to test for nonparametric data, including parity, number of vaginal births and duration of hospital stay. Fisher’s exact was used to test for differences in categorical variables, such as smoking status, co-morbidities, preoperative symptoms, preoperative management, complications and PGI-I. Significance was considered when *p < 0.05*.

## Results

**Fig. 1:**
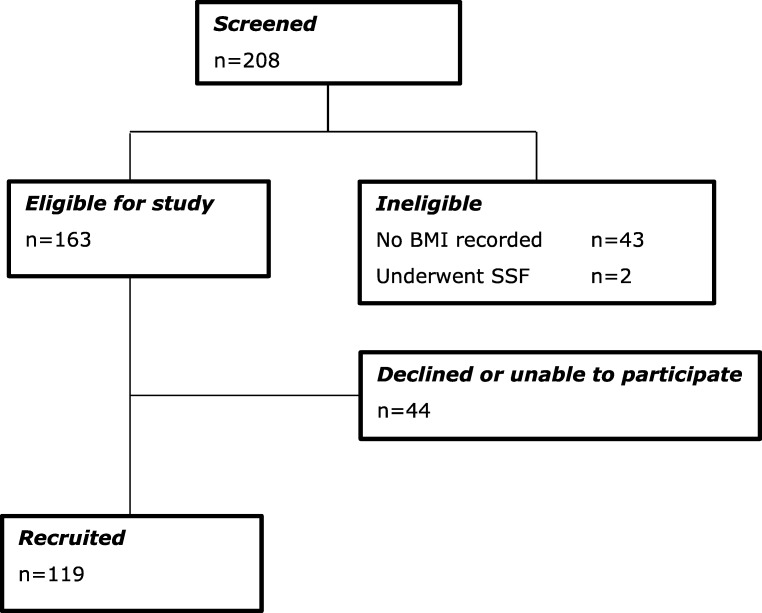
Participant flow through the study

Between March 2005 and January 2013, 208 women were listed for LSC. During this time LSC was offered as primary management for vault prolapse, irrespective of BMI. Forty-five women were ineligible for the study: two had a sacrospinous fixation (SSF) for anaesthetic considerations, and 43 had no record of preoperative BMI in the surgical or anaesthetic notes. Of the 163 eligible women, 119 (73.0%) were recruited and provided informed consent; 44 (26.0%) declined to participate in the study, were unable to provide informed consent or were unable to attend for follow-up. This study reports on data of 119 consecutive women (Fig. 1). Median follow-up was 6 months [interquartile range (IQR) 5–7 months]. Participant demographics, including distribution according to BMI for each group, are listed in Table 1.

**Table 1:** Baseline demographics of participants by normal, overweight and obese BMI class

	BMI class			*P* value
	normal weight	Overweight	Obese	
BMI Median, (IQR)	23.2, (22.2–24.4) *n* = 39	26.9 (26.0–28.1) *n* = 54	32.4, (31.5–33.7) *n* = 26	<0.001
Age Median, (IQR)	60 (54–67)	63.5 (57–67)	61.5 (58–67)	0.618
Parity Median (IQR)	2, (2–3)	2, (2–3)	2, (2–3)	0.982
Vaginal births Median (IQR)	2, (2–3)	2, (2–3)	2, (2–3)	0.951
Smoker Frequency (%)	8 (22.86)	7 (13.64)	3 (13.73)	0.527
Cormobidities, frequency (%)				
All	8 (20.5)	10 (18.5)	4 (15.4)	0.953
Diabetes	2 (5.1)	2 (3.7)	0	0.817
Cardiovascular	6 (15.4)	7 (13.0)	4 (15.4)	0.891
Respiratory	0	3 (5.6)	0	0.314
Previous laparotomy	22 (56.4)	28 (51.9)	15 (57.7)	0.942
Conservative management, frequency (%)				
Physiotherapy	14 (35.9)	17 (31.5)	9 (34.6)	0.899
Pessary	20 (51.3)	28 (51.9)	15 (34.6)	0.859
Previous prolapse surgery, frequecy (%)				
Laparoscopic sacrocolpopexy	1 (2.7)	3 (5.6)	0	0.553
Open sacrocolpopexy	1 (2.7)	5 (9.3)	2 (7.7)	0.507
Sacrospinous fixation	5 (12.8)	4 (7.4)	3 (11.5)	0.673

*BMI* body mass index, *IQR* interquartile range

The obese group included two women who were classified as morbidly obese. All women reported similar preoperative symptoms regardless of BMI group. There was no difference in preoperative conservative management with supervised pelvic floor exercises or pessary use in previous vault prolapse or continence surgery between the three groups. Obese women were more likely to have a preoperative stage 3–4 vault prolapse than those in the normal weight and overweight groups, which may reflect patient selection bias (Table 2). Some women presented with prolapse in more than one compartment.

**Table 2:** Preoperative symptoms and examination findings by normal, overweight and obese BMI class

	BMI class			*P* value, Fisher’s exact
	Normal	Overweight	Obese	
Symptoms, frequency (%)				
Bulge	38 (97.4)	53 (98.1)	25 (96.2)	0.796
Dragging sensation	3 (7.7)	5 (9.3)	5 (19.2)	0.304
Discomfort	7 (17.9)	15 (27.8)	7 (26.9)	0.562
Dyspareunia	6 (15.4)	6 (11.1)	4 (15.4)	0.829
Defecatory dysfunction	6 (15.4)	7 (13.0)	4 (15.4)	0.891
Voiding dysfunction	8 (20.5)	7 (13.0)	7 (26.9)	0.297
POP-Q, frequency (%)				
Anterior compartment				
Stage 0–2	19 (54.3)	28 (56.0)	8 (36.4)	0.181
Stage 3–4	16 (45.7)	22 (44.0)	14 (63.6)	
Posterior compartment				
Stage 0–2	26 (74.3)	26 (54.2)	11 (50.0)	0.263
Stage 3–4	9 (25.7)	22 (45.8)	11 (50.0)	
Apical compartment				
Stage 1	8 (22.8)	7 (14.3)	0	0.017^*^
Stage 2	21 (60.0)	32 (65.3)	11 (50.0)	
Stage 3–4	6 (17.1)	10 (20.4)	11 (50.0)	

Missing data from case records for entire POP-Q in four women in the normal, eight in the overweight and five in the obese group; for posterior compartment for two women in the overweight group; for apical compartment in one woman in the overweight group. Some women presented with prolapse in more than one compartment. Vault POP defined as point C at stage 2, or stage 1 with a large concomitant anterior- or posterior-compartment POP

*BMI* body mass index; *BMI class* normal 18.5–24.9 ( *n* = 39), overweight 25.0–29.9 (*n* = 54), obese ≥30.0 (*n* = 26), *POP* pelvic organ prolapse, *POP-Q* Pelvic Organ Prolapse Quantification system

### Primary outcome

There were no cases of conversion to laparotomy, vascular injury, blood loss requiring blood transfusion, return to theatre, deep vein thrombosis or wound infection (Table 3). Bladder injury occurred in two normal weight and two overweight women. All were closed intraoperatively with completion of the LSC and managed with an indwelling catheter for 1 week, with no long-standing morbidity. There was one ureteric injury in the normal weight group, which was successfully repaired at the time of LSC. One serosal rectal injury occurred in the normal-weight group, was recognised and oversewn intraoperatively with completion of the LSC, with no further complications or long-standing morbidity.

**Table 3:** Intra- and postoperative complications by normal, overweight and obese BMI class

Complication Frequency (%)	BMI class			*P* value, Fisher’s exact
	Normal	Overweight	Obese	
Injury				
Bladder	2 (5.1)	2 (3.7)	0	0.835
Bowel	1 (2.6)	0	0	0.548
Ureteric	1 (2.6)	0	0	0.548
Vascular	0	0	0	–
Conversion to laparotomy	0	0	0	–
Anaesthetic	4 (10.3)	2 (3.7)	1 (3.8)	0.414
Return to theatre				
24 h	0	0	0	–
7 days	0	0	0	–
Blood transfusion	0	0	0	–
Deep vein thrombosis	0	0	0	–
Urinary tract infection	1 (2.6)	1 (1.9)	0	1.0
Wound infection	0	0	0	–

*BMI* body mass index, *BMI class* normal 18.5–24.9 (*n* =  39), overweight 25.0–29.9 (*n* =  54), obese 30.0–39.9 (*n* =  24), morbidly obese ≥40.0

**Fig. 2:**
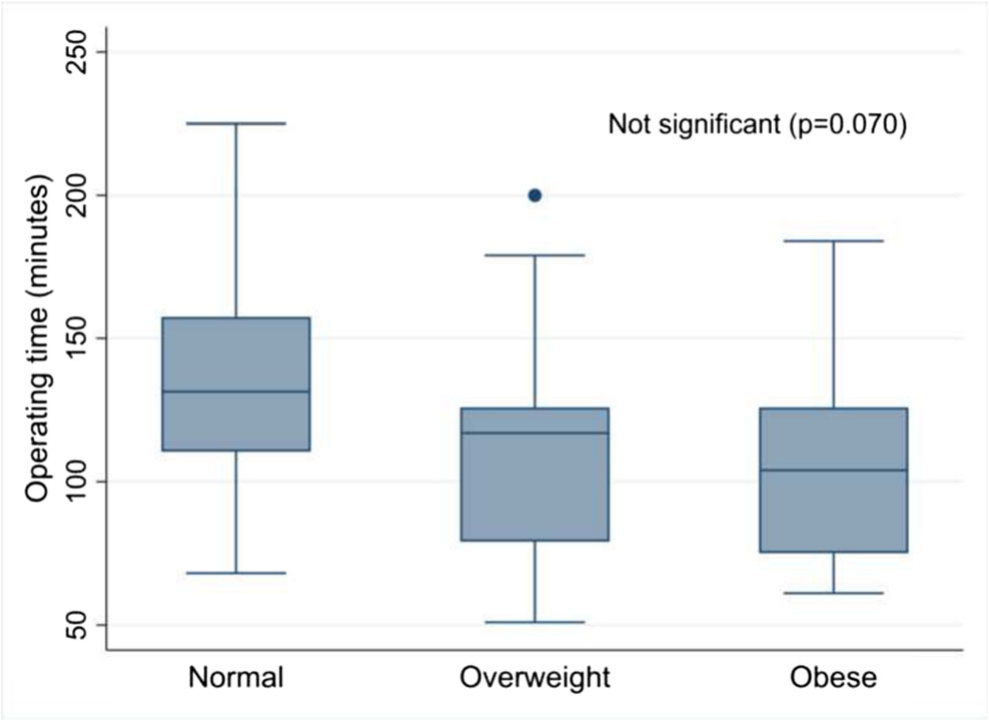
Operating time by normal, overweight and obese body mass index (BMI) class

**Fig. 3:**
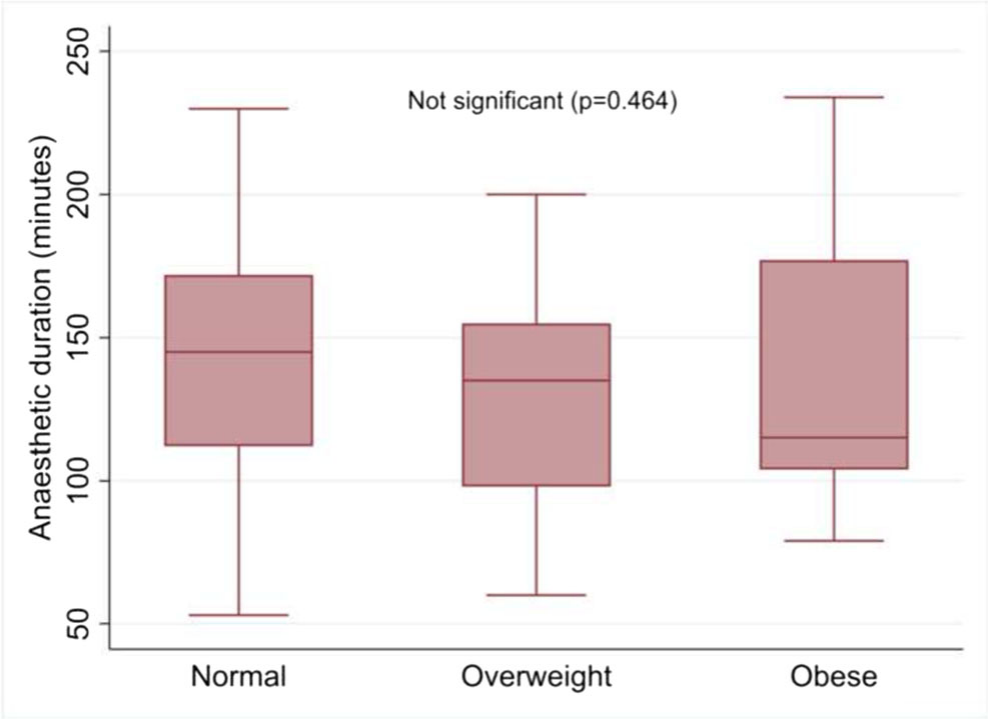
Duration of anaesthetic by normal, overweight and obese body mass index (BMI) class

There was a trend towards increased operating time in women of normal weight, although this was not statistically significant and not related to grade of lead surgeon (*p = 0.978*) (Fig. 2). There was no difference in duration of anaesthetic administration between groups (Fig. 3) or in duration of hospital stay (*p = 0.898;* median for all groups 1 day, IQR 1–2 days).

There were no cases of mesh erosion or other mesh complications.

### Secondary outcomes

Six-month follow-up data was available for 80 women. There was no significant difference in subjective cure rate, defined as very much or much better, on PGI-I. Rates were reported as 76.9% in the normal weight, 72% in the overweight and 65.4% in the obese group (overall *p = 0.669*, normal weight vs obese *p = 0.556*). Two women in the overweight group reported feeling very much worse: one developed chronic pelvic pain due to nerve entrapment following degenerative spinal disease, and one developed severe constipation.

There was no difference in objective cure at 6 months measured using the POP-Q for anterior, posterior and apical compartments between normal weight, overweight and obese women, (*p = 0.098, 0.282* and *0.402*, respectively). The apical compartment was above the level of the midvagina (defined as half the total vaginal length) in all women except for one case of recurrence. This recurrence occurred in the overweight group in woman who experienced a stage 3 vault POP, which she reported occurred suddenly 5 months postoperatively following a chest infection. She subsequently declined further investigation and was treated with a ring pessary.

## Discussion

This study was a secondary analysis to evaluate outcomes and safety of LSC in obese, overweight and normal weight women. The results demonstrate no significant difference in subjective or objective cure rate, perioperative complications, operating time, duration of anaesthetic or hospital stay. Despite the clinical need for such data, only one other study, that by Thubert et al., has compared outcomes and safety of LSC in obese and nonobese women in the medium term. The authors also found no significant difference in outcomes, complications or patient satisfaction between obese and nonobese women 2 months postoperatively [8]. However, their data combined normal weight and overweight women into one larger group with a shorter follow-up time of 2 months. They also reported a conversion to laparotomy rate due to vascular injury of 5% in obese women, whereas there were no cases of vascular injury or conversion to laparotomy in our study.

A study by Turner et al. evaluated complication rates and outcomes in normal weight, overweight and obese women undergoing both robotic sacrocolpopexy (RSC) and LSC reported that obese women were more likely to undergo RSC (*p = 0.004*) [13]. They found increased blood loss in obese women (*p = 0.003*), although total blood loss was not clinically significant, at <100 ml, for all groups, which is in keeping with our findings. Turner et al. also reported longer operating times in obese compared with normal weight and overweight women (*p = 0.003*; 234 min normal weight and overweight; 251 min obese). These operating times are significantly longer than those described in our study (129 min normal weight, 112 min overweight, 107 min obese) and may reflect the inclusion of women undergoing concomitant hysterectomy and salpingo-oophorectomy, as well as the longer operating time associated with RSC [14, 15].

A study by Kissane et al. evaluated outcomes in RSC and reported significantly longer operating times of 202, 206 and 216 min in normal weight, overweight and obese women, respectively, when compared with our cohort [16]. Again, this may reflect the high incidence of concomitant surgery and longer operating time of RSC in their study.

Halder et al. compared LSC, including RSC, to ASC and found minimally invasive sacrocolpopexy was associated with longer operating times but fewer complications and shorter length of hospital stay for normal weight, overweight and obese women, suggesting overweight and obese women should be offered either LSC or RSC for vault prolapse [17].

Our study provides further data supporting LSC for managing vault prolapse in obese women. We stratified the report using the WHO classification to provide greater clarity on the impact of BMI range.

### Limitations

This was a single-site, retrospective study which may have led to selection bias. However, this was minimised by the fact all women with vault prolapse were offered LSC during this time frame, regardless of BMI. The retrospective design of the study also meant there were some missing data points. Due to the size of the obese cohort, data for these women were combined into one group rather than being analysed separately obese, severely obese and morbidly obese women. Morbidly obese women, in particular, present a different set of surgical and anaesthetic challenges to obese and severely obese women, meaning conclusions from this study should be extrapolated to morbidly obese women with caution.

Although the three groups had similar baseline characteristics, the obese cohort was more likely to have a stage 3–4 apical POP on preoperative POP-Q. This may reflect that obese women with stage 2 vault POP were less likely to opt for surgery or an abdominal approach, or that in this cohort, they presented with a greater degree of POP than women who were overweight or of normal BMI. Preoperative POP stage 3 or 4 is a risk factor for recurrence, and this bias could have affected subjective and objective cure rates in the obese group, although our analysis would suggest this was not the case [3, 18].

### Interpretation

This study demonstrates LSC is equally effective and with a comparable safety profile for normal weight, overweight and obese women when performed by a trained laparoscopic urogynaecologist. Data show a trend towards increased operating time in normal weight women. One possible explanation is that surgeons are more likely to undertake complex cases, such as those with dense adhesions or previous urostomy, in women of normal BMI. An alternative explanation is that surgeons may prefer teaching trainee surgeons whilst operating on women of normal BMI, which in turn may lead to longer operating times. This could also be a reflection of the small sample size.

### Generalisability

All operations were performed in a tertiary unit by subspecialist urogynaecologists trained in laparoscopic urogynaecology. LSC can be technically challenging and requires an experienced laparoscopic surgeon to perform, particularly in overweight and obese women due to difficulty accessing the sacral promontory. This is because the sacral promontory may be more difficult to identify and dissect in overweight and obese women, increasing the risk of a major vascular injury. Another difficulty performing LSC in obese women is in maintaining adequate ventilation while generating sufficient abdominal pressure to perform the procedure. We found this is easier when working with anaesthetists with bariatric experience. Our findings may not be generalisable to smaller units that do not have the equipment or personnel for managing obese women undergoing laparoscopy.

### Overall

Findings from this study are important, particularly in the context of the expanding obesity epidemic. Our data supports offering LSC to obese women with vault prolapse. It is our practice to offer both a LSC and a non-mesh alternative in the form of a vaginal sacrospinous fixation to all suitable women presenting with vault prolapse who wish to have surgical management as part of a patient-centred, nondirective approach to care.
